# The Effectiveness of Synbiotic on the Improvement of Clinical Symptoms in Children with Eosinophilic Esophagitis

**DOI:** 10.1155/2022/4211626

**Published:** 2022-03-07

**Authors:** Niloufar Amini, Majid Khademian, Tooba Momen, Hossein Saneian, Peiman Nasri, Fatemeh Famouri, Giti Ebrahimi

**Affiliations:** ^1^Child Growth and Development Research Center, Research Institute for Primordial Prevention of Non-Communicable Disease, Isfahan University of Medical Sciences, Isfahan, Iran; ^2^Metabolic Liver Disease Research Center, Isfahan University of Medical Sciences, Child Growth and Development Research Center, Research Institute of Primordial Prevention of Non-Communicable Disease, Isfahan University of Medical Sciences, Isfahan, Iran; ^3^Department of Asthma, Allergy and Clinical Immunology, Child Growth and Development Research Center, Research Institute of Primordial Prevention of Non-Communicable Disease, Isfahan University of Medical Sciences, Isfahan, Iran; ^4^Pediatrics Department, School of Medicine, Isfahan University of Medical Sciences, Isfahan, Iran

## Abstract

**Background:**

Eosinophilic esophagitis (EoE) is an allergic inflammatory disorder of the esophagus. Today, probiotics are included as adjuvant therapy in the treatment of allergic diseases. The aim of this study was to assess the effect of synbiotic on clinical symptom improvement in EoE patients.

**Methods:**

This study is designed by a double-blind, placebo-controlled clinical trial with two parallel groups, which was performed on 30 children with eosinophilic esophagitis. All participants were children aged 6 months to 15 years. Both groups received the same treatment (elimination diet, topical steroid, and proton pump inhibitor). A synbiotic (KidiLact) was added to the medication regimen of 15 patients (case), while the next 15 patients received a placebo (control). Severity and frequency of symptoms were assessed with a checklist derived from a validated scoring tool in both groups before and after 8 weeks of treatment.

**Results:**

There was a significant reduction in the severity score of chest pain and poor appetite (*P* value < 0.05) in the case group taking probiotics, while nausea and poor appetite were the only symptoms with a significant reduction in the frequency score after intervention in this group.

**Conclusion:**

Probiotics can be used as adjuvant treatment for patients with EoE. Improvement in the severity of chest pain and poor appetite and reduction in the frequency of nausea and poor appetite in these patients can be seen.

## 1. Introduction

Eosinophilic esophagitis (EoE) is a chronic inflammatory disorder of the esophagus that manifests as esophageal dysfunction and histologically with increasing eosinophil in esophageal tissue [1, 2]. The symptoms are variable including vomiting, regurgitation, and growth retardation in infants and in older children as heartburn, dysphagia, and abdominal pain [3]. The prevalence of this disease is estimated at 0.5–1 in 1000. EoE could be seen in 2–7% of patients undergoing endoscopy for any reason and 12–23% undergoing endoscopy for dysphagia [3].

Little is known about the incidence of EoE, but based on recent data, it seems that the incidence of EoE has increased during the last decade, which might be mainly due to early diagnosis [4]. The underlying cause of eosinophilic esophagitis is still unknown; it may be due to increased sensitivity to food and aeroallergens [5].

Studies show that there could be a strong association between EoE and other allergic and atopic disorders. Most of the patients with EoE are sensitive to various food allergens, which indicates the background of the allergic disease [6, 7]. The role of probiotics in other allergic diseases, including atopic dermatitis, asthma, eosinophilic gastroenteritis, and allergic IBS, has been discussed in various studies, and positive effects have been reported [8–10].

Therefore, it seems that probiotic administration along with the main treatments of allergic disease can be an effective treatment strategy in improving the symptoms by creating a sufficient balance between intestinal bacteria. Probiotics are safe, noninvasive, noncarcinogenic, and nonpathogenic bacteria or yeast [11]. Probiotic bacteria belong mainly to the group Lactobacillus and Bifidobacterium, especially Lactobacillus acidophilus and Bifidobacterium bifidum. Some common probiotics, such as Saccharomyces boulardii, are yeast.

As mentioned above, EoE is an important condition for which no definite therapeutic strategy has been yet discovered. Due to this issue and considering the possible effective roles of probiotics in improving symptoms in EoE patients, in this study, we aimed to investigate the roles of probiotics in EoE.

## 2. Materials and Methods

This clinical trial was performed in 2019-2020 in the Pediatric Imam Hossein Hospital affiliated with Isfahan University of Medical Sciences. The current study was conducted on 30 children with confirmed eosinophilic esophagitis. The study protocol was approved by the Research Committee of Isfahan University of Medical Sciences, and the ethics committee has confirmed it (ethics code: IR.MUI.MED.REC.1398.365, Iranian Registry of Clinical Trials (IRCT) code: IRCT20171230038142N14).

The inclusion criteria were children aged between 6 months and 15 years; referral to pediatric gastroenterologists due to complaints such as vomiting, eating problems, chest or epigastric pain, and drooling; poor response to at least 4 weeks' treatment by an appropriate dose of proton pump inhibitor medications (1 mg/kg/day); diagnosis of EoE based on pathologic studies; and signed written informed consent by parents to participate in this study. The exclusion criteria were lack of proper follow-up, lack of drug compliance, and patient's will to exit the study.

Before entering the study, all cases underwent esophagogastroduodenoscopy. At least three individual biopsy specimens were obtained from the esophagus. Two biopsies were taken from the distal esophagus and one from the midesophagus; also, biopsies from the duodenum and stomach were taken to rule out eosinophilic gastroenteritis. Cases with endoscopic findings compatible with EoE such as furrowing, trachealization, whitish exudate, and more than 15 eosinophils in HPF in microscopy of esophageal samples were diagnosed as EoE. All cases had no significant eosinophil count in gastric and duodenal samples.

After recruiting the study samples, the patients were randomly divided into two groups of intervention and control, based on the pairing and individuality of the national code. Before starting the treatments, demographic variables, chief complaint, history, and physical examination of patients were recorded in the appropriate files. We used a checklist derived from the Pediatric Eosinophilic Esophagitis Symptom Score (PEESS v2.0) that was reviewed and confirmed by expert pediatric gastroenterologists [12, 13], to assess the frequency and severity of EoE-related symptoms. This checklist consists of 18 items, nine of which are related to frequency and the rest to the severity of symptoms. The answers to each item are distributed on a scale with scores ranging from 0 to 4. In items referring to frequency, 0 corresponds to “never” and 4 corresponds to “almost always” (two or more times a day). In items referring to severity, 0 corresponds to “not bad” and 4 corresponds to “very bad.” The higher scores indicate higher frequency of symptoms or/and more severe symptoms.

Both groups were matched in terms of age, sex, clinical symptoms, elimination diet, and medications. Both groups were pretreated with PPI, and both groups underwent diet modification treatments and topical steroids including fluticasone spray. Along with the mentioned therapeutic actions, the first group of patients was treated with KidiLact sachet (a synbiotic from the Iranian Bio-Fermentation Pharmaceutical Company) with a daily dose of two for 8 weeks and the second group received a placebo with an appearance similar to the KidiLact sachet.

KidiLact product contains 10^9^ beneficial bacterial strains, including Lactobacillus casei, Lactobacillus acidophilus, Lactobacillus rhamnosus, Lactobacillus bulgaricus, Bifidobacterium infantis, Bifidobacterium breve, and Streptococcus thermophilus.

Patients were visited every two weeks, and the correct use of the drug was ensured. After 8 weeks, the patients were evaluated and compared again through the checklist of their clinical symptoms, and results were compared with the results before the intervention.

The obtained data were entered into the Statistical Package for the Social Sciences (SPSS) (version 24, SPSS Inc., Chicago, IL). Quantitative data were reported as the mean ± standard deviation and qualitative data as frequency distribution (percentage). The independent *t*-test and chi square were used to analyze the data. *P* value < 0.05 was considered a significance threshold.

## 3. Results

**Figure 1:**
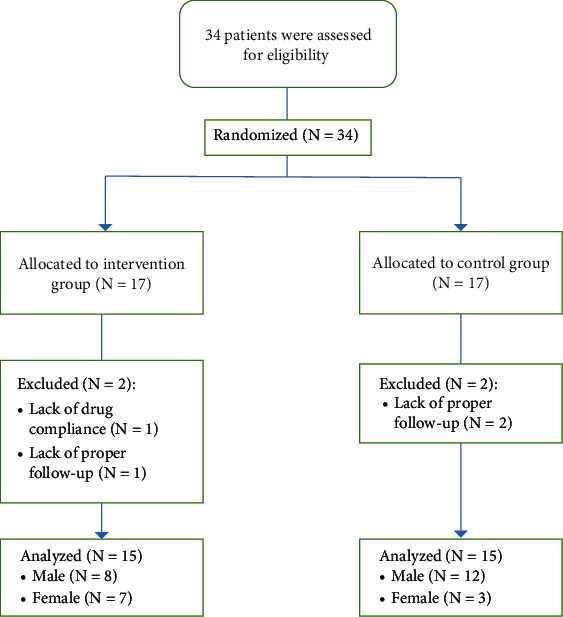
The CONSORT flow chart of the study.

In this study, 34 patients with the diagnosis of EoE entered the study and were divided into two groups each containing 17 patients. During the interventions, 4 patients (two in each group) were excluded due to lack of proper follow-up (*N* = 3) and lack of drug compliance (*N* = 1). At the end, data of 30 patients were analyzed. The CONSORT flow chart of the study is shown in Figure 1.

The study population consisted of 20 boys (66.6%) and 10 girls (33.4%). The mean age of participants in the case group was 6.33 ± 1.82 years and in the control group was 6.78 ± 2.69 years. There were no significant differences between the case and control groups regarding age and gender (*P* = 0.619 and 0.999, respectively).

Evaluation of the severity of patients' symptoms in this study demonstrated a significant decrease in total severity scores in both the case (*P* = 0.002) and control (*P* = 0.001) groups. Furthermore, we observed a significant decrease in nausea, vomiting, and poor appetite in both groups (*P* < 0.05 for all). Patients in the intervention group had significant improvements in dysphagia (*P* = 0.039), and patients in the control group had significant improvements in stomach ache (*P* = 0.001) and reflux (0.038). These data are shown in Table 1.

The mean ± standard deviation of the total frequency score before the intervention in the control group was 6.44 ± 2.36 and in the case group was equal to 7.08 ± 2.74. This value after the intervention in the control and case groups was 1.56 ± 0.98 and 1.0 ± 0.75, respectively. The mean of the total frequency score of symptoms in patients after the intervention decreased significantly in both the control and case groups (*P* value < 0.05).

Table 2 indicates the frequency score of symptoms before and after the intervention between the case and control groups. After intervention, nausea and poor appetite were the only symptoms with a significant reduction in the frequency score in the case group.

## 4. Discussion

EoE is an eosinophil-rich, Th2 antigen-mediated disease of increasing pediatric and adult worldwide prevalence. Diagnosis requires greater than or equal to 15 eosinophils per high-power field on light microscopy [15, 16]. On the other hand, EoE is an allergic inflammatory disease that is triggered by food allergens and characterized by progressive esophageal dysfunction.

Gut microbiota plays a beneficial role in food digestion, development of the immune system, control/growth of the intestinal epithelial cells, and their differentiation [17]. Dysregulation of gut microbiota (dysbiosis) has also been found to be associated with an increased risk of allergies [18]. Prescribing probiotics causes a significant change in the intestinal microflora and modulates cytokine secretion and increased intestinal IgA responses. The modulation of the T helper cell (Th1/Th2) balance is done by probiotics [15]. In general, probiotics are associated with a decrease in inflammation by increasing butyrate production and induction of tolerance with an increase in the ratio of cytokines such as IL-10/IFN-*γ*, Treg/TGF-*β*, reducing serum eosinophil levels, and the expression of metalloproteinase-9 which contribute to the improvement of the allergic disease's symptoms [17, 18].

Therefore, probiotic administration along with the main treatments of allergic disease can be effective in improving the symptoms by creating a sufficient balance between intestinal bacteria. Probiotics are safe, noninvasive, noncarcinogenic, and nonpathogenic [11] and include bacteria or yeast. Probiotic bacteria belong mainly to the group Lactobacillus and Bifidobacterium, especially Lactobacillus acidophilus and Bifidobacterium bifidum. Some common probiotics, such as Saccharomyces boulardii, are yeast. The findings of this study were compared with other studies and are most likely similar to them.

In 2016, a similar study was performed by Holvoet and others in a murine model of EoE. In this EoE model, supplementation with Lactococcus lactis NCC 2287 significantly decreased esophageal and bronchoalveolar eosinophilia. This was the first study in an animal model to show that a specific probiotic could affect eosinophilic esophagitis, and this was suggested for the treatment of humans with esophagitis [19].

In another study, Kryuchko et al. showed that the use of probiotics is effective in improving the symptoms of eosinophilic gastroenteritis as well as the manifestations of atopic dermatitis [8]. Also in the United States, Martin et al. showed an improvement in the symptoms of allergic proctocolitis following probiotic use [9].

de Kivit and colleagues showed improvement in the symptoms of allergic gastroenteritis (including eosinophilic gastroenteritis and allergic IBS) after a 4-week period of symbiotic use [10]. The results of these studies are consistent with the results of our studies on the improvement of clinical symptoms in patients following probiotic use. In another study, probiotic administration in late infancy showed a significantly lower incidence of eczema in the probiotic group compared to the placebo group [20].

A meta-analysis was conducted in 2019 that showed that probiotic supplementation during both the prenatal and postnatal periods reduced the incidence of AD in infants and children. These findings suggest that starting probiotic treatment during gestation and continuing through the first 6 months of the infant's life may be of benefit in the prevention of AD [14].

Another study was performed in 2018 by Sharma and Im. They reported that probiotics could be used as a drug to modulate the immune system and the therapeutic target recommended for the treatment of allergic diseases such as atopic dermatitis and food allergies [18].

Based on a study by Krzych-Fałta and others in 2018, the use of probiotics in the Polish population showed no protective effect on any of the evaluated disorders in early childhood. Conversely, over the age of 14 years, probiotic formulations exhibit health-promoting effects and may lower the risk of allergic diseases [21].

In this study, we investigated the effect of synbiotic on the improvement of clinical symptoms in these patients, and it is noteworthy that no such study has been performed on these patients.

The findings of this study were compared with other studies and are most likely similar to them.

It is observed that in the case group, those taking probiotics, the total severity score of symptoms decreased significantly after treatment. After the intervention, the severity score of chest pain was different significantly between the case and control groups. Also, the mean of total frequency score of symptoms in patients after the intervention decreased significantly in both the control and case groups.

The limitations of the current study were the restricted study population and the non-revaluation of the pathologic findings after the treatments due to the invasiveness of endoscopy.

## 5. Conclusion

The present study shows that adjuvant therapy with probiotics may improve the severity of chest pain and poor appetite and reduce the frequency of nausea and poor appetite in EoE patients.

## Figures and Tables

**Table 1:** Comparison of severity scores pre- and postintervention between groups.

		Case (*n* = 15)		Control (*n* = 15)		*P* value^∗^
		Median (IQR)^#^	[Min, Max]	Median (IQR)	[Min, Max]	
Total score of severity	Before	14 (6.5)	[6, 28]	12 (6.5)	[6, 14]	0.309
	After	2 (1.5)	[0, 4]	4 (2.0)	[0, 8]	0.221
	*P* value^∗∗^	0.002		0.001		
Chest pain	Before	0 (0.0)	[0, 50]		[0, 25]	0.655
	After	0 (0.0)	[0, 25]	0 (0.0)	[0, 0]	0.020^∗^
	*P* value^∗∗^	0.317		0.317		
Nausea	Before	25 (50.0)	[0, 75]	50 (50.0)	[0, 75]	0.981
	After	0 (0.0)	[0, 25]	12.5 (25.0)	[0, 25]	0.143
	*P* value^∗∗^	0.026		0.002		
Vomiting	Before	12.5 (56.25)	[0, 100]	25 (50.0)	[0, 75]	0.280
	After	0 (0.0)	[0, 0]	0 (0.0)	[0, 25]	0.999
	*P* value^∗∗^	0.042		0.004		
Stomach ache	Before	0 (50.0)	[0, 75]	25 (31.25)	[0, 75]	0.280
	After	0 (25.0)	[0, 25]	0 (25.0)	[0, 25]	0.999
	*P* value^∗∗^	0.098		0.001		
Poor appetite	Before	50 (50.0)	[0, 75]	25 (50.0)	[0, 50]	0.038
	After	0 (0.0)	[0, 25]	0 (25.0)	[0, 25]	0.037^∗^
	*P* value^∗∗^	0.004		0.008		
Dysphagia	Before	0 (25.0)	[0, 75]	0 (6.25)	[0, 50]	0.320
	After	0 (0.0)	[0, 0]	0 (0.0)	[0, 0]	0.999
	*P* value^∗∗^	0.039		0.059		
Reflux	Before	0 (0.0)	[0, 25]	0 (25)	[0, 50]	0.255
	After	0 (0.0)	[0, 0]	0 (0.0)	[0, 0]	0.999
	*P* value^∗∗^	0.317		0.038		
Sore throat	Before	0 (0.0)	[0, 0]	0 (0.0)	[0, 50]	0.201
	After	0 (0.0)	[0, 0]	0 (0.0)	[0, 0]	0.999
	*P* value^∗∗^	0.317		0.999		
Need to drink fluids	Before	0 (25.0)	[0, 100]	0 (0.0)	[0, 50]	0.465
	After	0 (0.0)	[0, 0]	0 (0.0)	[0, 25]	0.078
	*P* value^∗∗^	0.109		0.157		

^∗^Resulted from the comparison of between-group (case-control) by independent *t*-test. ^∗∗^Resulted from the comparison of within-group (before-after) by paired *t*-test. ^#^Interquartile range (IQR).

**Table 2:** Comparison of frequency score pre- and postintervention between groups.

		Case (*n* = 15)	Control (*n* = 15)	*P* value^∗^
		Frequency score (%)	Frequency score (%)	
Chest pain	Before	5 (26.3)	14 (73.7)	0.041
	After	1 (100.0)	0 (0.0)	0.400
	*P* value^∗∗^	0.125	0.001^∗^	
Nausea	Before	7 (36.8)	12 (63.2)	0.643
	After	1 (10.0)	9 (90.0)	0.024^∗^
	*P* value^∗∗^	0.031^∗^	0.250	
Vomiting	Before	5 (33.3)	10 (66.7)	0.456
	After	0 (0.0)	3 (100.0)	0.255
	*P* value^∗∗^	0.063	0.016^∗^	
Stomach ache	Before	5 (45.5)	6 (54.5)	0.280
	After	3 (33.3)	6 (66.7)	0.704
	*P* value^∗∗^	0.500	0.999	
Poor appetite	Before	11 (50.0)	11 (50.0)	0.064
	After	1 (11.1)	8 (88.9)	0.042^∗^
	*P* value^∗∗^	0.002^∗^	0.250	
Dysphagia	Before	5 (55.6)	4 (44.4)	0.255
	After	0 (0.0)	0 (0.0)	—
	*P* value^∗∗^	0.063	0.125	
Reflux	Before	1 (16.7)	5 (83.3)	0.255
	After	0 (0.0)	0 (0.0)	—
	*P* value^∗∗^	0.999	0.063	
Sore throat	Before	1 (100.0)	0 (0.0)	0.400
	After	0 (0.0)	0 (0.0)	—
	*P* value^∗∗^	0.999	—	
Need to drink fluids	Before	3 (60.0)	2 (40.0)	0.317
	After	2 (100.0)	0 (0.0)	0.152
	*P* value^∗∗^	0.999	0.999	

^∗^Resulted from the comparison of between-group (case-control) by chi square. ^∗∗^Result from the comparison of within-group (before-after) by Mcnemar test.

## Data Availability

This paper is extracted from an MD thesis at Isfahan University of Medical Sciences, and the underlying data supporting the results of this study can be found at the Research Center of the Isfahan School of Medical Sciences.
